# Nivolumab Combined With Ipilimumab Treatment Induced Hypophysitis and Immune-Mediated Liver Injury in Advanced Esophageal Squamous Cell Carcinoma: A Case Report

**DOI:** 10.3389/fonc.2022.801924

**Published:** 2022-04-01

**Authors:** Yi Feng, Chengyang Li, Yuan Ji, Ying Liu, Lu Gan, Yiyi Yu, Tianshu Liu

**Affiliations:** ^1^ Department of Medical Oncology, Zhongshan Hospital, Fudan University, Shanghai, China; ^2^ Center of Evidence-Based Medicine, Fudan University, Shanghai, China; ^3^ Cancer Center, Zhongshan Hospital, Fudan University, Shanghai, China; ^4^ Clinical Pharmacology, The Ohio State University, Columbus, OH, United States; ^5^ Department of Pathology, Zhongshan Hospital, Fudan University, Shanghai, China; ^6^ Department of Clinical Coordination, Hangzhou Simo Co., Ltd., Hangzhou, China

**Keywords:** immune checkpoint inhibitor, immune-related adverse events, hypophysitis, immune-mediated liver injury, steroid therapy

## Abstract

Immune checkpoint inhibitors (ICIs) have transformed the treatment in malignancies because of the impact on reactivating the immune cells to kill tumor cells. Because anti-CTLA-4 antibody and anti-PD-1 antibody (or anti-PD-L1 antibody) work in different ways, they have synergistic effects when used in combination in many cancers. However, it has been found that a strong immune response may lead to more serious and multi-system immune-related adverse events (irAE). We describe an advanced esophageal squamous cell carcinoma patient who received nivolumab combined with ipilimumab resulting in hypophysitis and immune-mediated liver injury. He was enrolled into a CheckMate 648 global, multicenter, randomized phase 3 Clinical Trial (CTR20171227) investigating the combined potency of nivolumab and ipilimumab in the treatment of patients with advanced esophageal squamous cell carcinoma and admitted to our center (site 0200). The patient developed hypophysitis and immune-related hepatitis rapidly after ICIs therapy, leading to the interruption of anti-tumor therapy. Then the patient developed Herpes zoster and recurrence of tuberculosis after treatment of irAEs with glucocorticoids. We report this case in the hope that doctors need to have sufficient knowledge and attention to the occurrence of irAE during the anti-immune combination therapy and actively intervene as soon as possible to obtain better anti-tumor effects and less harm to patients.

## Introduction

Cytotoxic T lymphocyte associated antigen-4 (CTLA-4) and programmed death-1 (PD-1) pathways are commonly involved in suppressing antitumor immune responses (1). In the tumor microenvironment, the expression of immunosuppressive molecules such as CTLA-4, PD-1 and its ligand PD-L1 (programmed death ligand 1), is significantly increased, resulting in apoptosis or inactivation of cytotoxic T lymphocytes, which allows tumor cells to escape the immune surveillance and clearance (2). Immune checkpoint inhibitors (ICIs) become a major development in cancer treatment because of the impact on reactivating immune cells. Ipilimumab (anti-CTLA-4 antibody), nivolumab (anti-PD-1 antibody), pembrolizumab (anti-PD-1 antibody), atezolizumab (anti-PD-L1 antibody), devaluzumab (anti-PD-L1 antibody), etc., are the ICIs approved for clinical cancer treatment. They certainly have a strong potency in many tumors. However, they also destroy the homeostasis of immune system and increase the risk of immune-related adverse events (irAEs). irAEs can affect all tissues and organs with different severity, mainly in the intestines, skin, endocrine glands, liver, lungs, etc. (1, 3). Khoja et al. summarized the irAE caused by different ICI treatments from 48 trials (6,938 patients), namely, 26 CTLA-4, 17 PD-1, 2 PD-L1 trials, and 3 studies tested by both CTLA-4 and PD-1. They found that different ICIs have distinct irAE profiles. Anti-CTLA-4 treatment has more possibility to induce grade 3/4 irAE (3). Appropriate treatment for irAE, therefore, is very important to avoid subsequent adverse events. Here, we describe an advanced esophageal squamous cell carcinoma patient who received nivolumab combined with ipilimumab resulting in hypophysitis and immune-mediated liver injury. He was enrolled onto a CheckMate 648 global, multicenter, randomized phase 3 Clinical Trial (CTR20171227) investigating the combined potency of nivolumab and ipilimumab in the treatment of patients with advanced esophageal squamous cell carcinoma and admitted to our center (site 0200). The patient developed herpes zoster and recurrence of tuberculosis after treatment of irAEs with glucocorticoids. We report this case to discuss the identification and intervention in the early stages of irAEs caused by cancer immunotherapy. We hope it will be helpful in diagnosis and the subsequent treatment of irAEs.

## Case

### Patient

**Figure 1:**
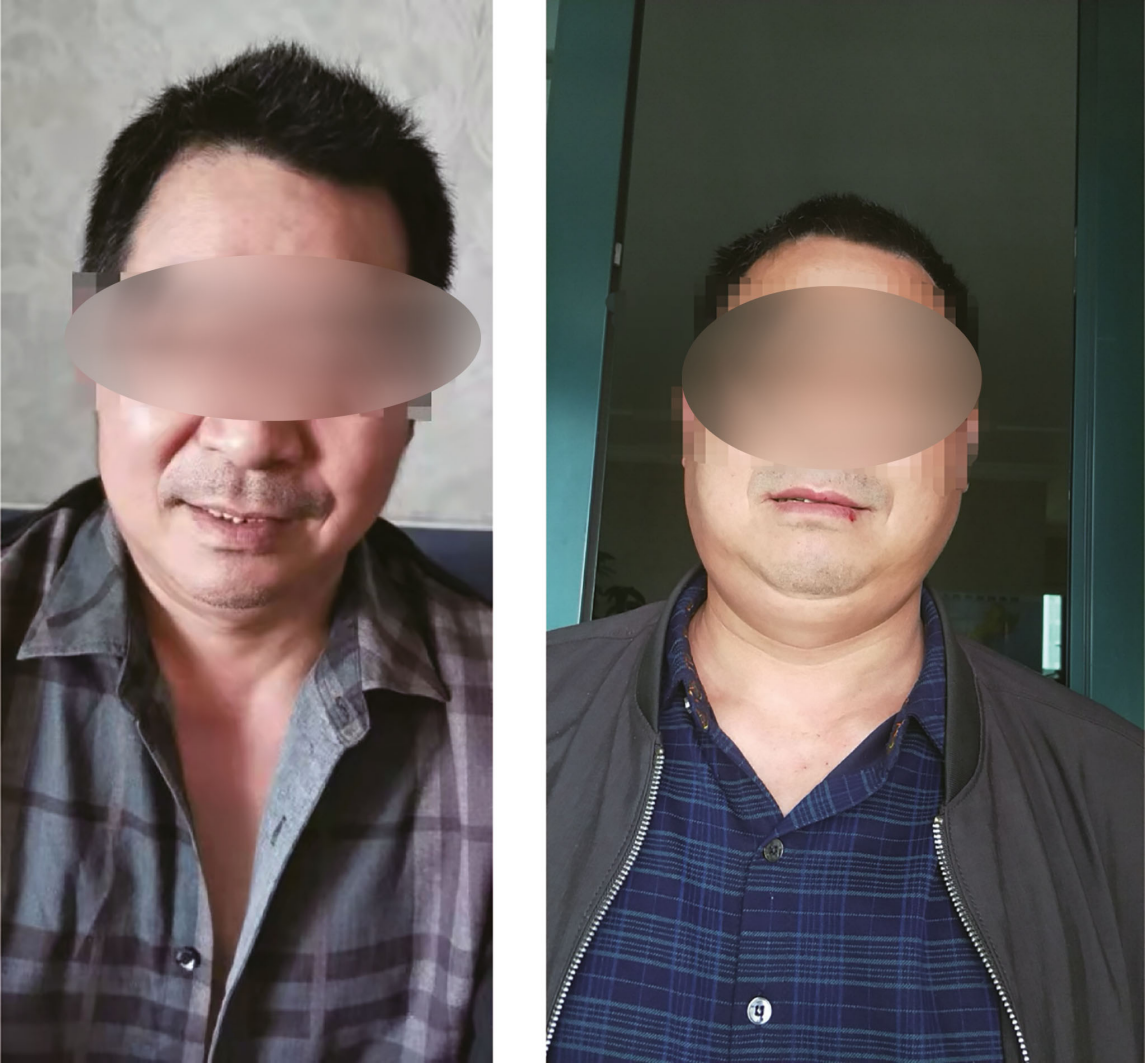
Patient pictures. Left picture: Before treatment; Right picture: One month after treatment.

A 55-year-old male patient was diagnosed with esophageal squamous cell carcinoma (cTxNxM1 stage IV lymph node) in March 2019. On 3 April 2019, he entered “an unresectable late-stage recurrent or metastatic esophageal squamous cell carcinoma that has not been treated before a randomized phase 3 study of nivolumab + ipilimumab or nivolumab combined with fluorouracil + cisplatin versus fluorouracil + cisplatin”. The patient was randomly assigned to group A (nivolumab + ipilimumab regimen). The first cycle of treatment started from 16 April 2019 with nivolumab (3 mg/kg) 204 mg dl, 15, 30 and ipilimumab (1 mg/kg) 68 mg dl, q6w. The first-week treatment was successfully completed. Previously the patient denied the history of hypertension, diabetes, coronary heart disease, liver disease or autoimmune diseases. Hepatitis B and hepatitis C antibodies were negative before being enrolled in the study. Tuberculosis was diagnosed in 2004, and he was cured after 8 months of anti-tuberculosis. T-SPOT (T-SPOT.TB, one type of interferon-gamma-release assay) and chest CT (computed tomography) were all negative before enrollment in the study. Skin itching (neck, face, back) began on 22 April 2019, with swelling on the face, ears to the lower neck on 4 May 2019, accompanied by dry mouth. On 20 May 2019, swelling occurred on the scalp, following by aggravating and spreading to the entire face and neck, which has affected the daily life of the patient. There was no swelling of the lower limbs and decrease in urine output. Physical examination revealed a small number of scattered rashes on the neck, face and back, obvious swelling on the face without depression, and restricted neck movement (**Figure 1**).

### Laboratory and Imaging Examinations

**Figure 2:**
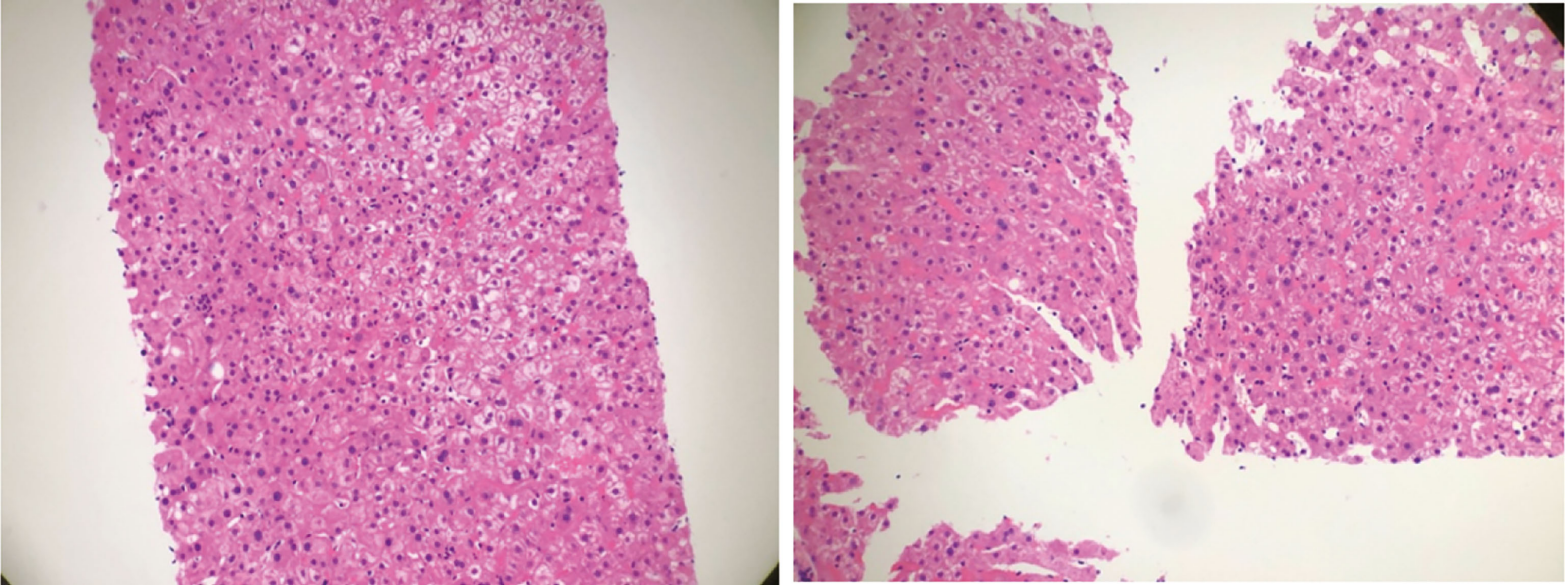
Hematoxylin–eosin staining of liver biopsy. It showed that the structure of liver lobules was intact. Part of liver cells was turbid and swollen. Mild congestion and lymphocytes were seen in the liver sinusoids. Neutrophil aggregation was seen in small foci.

30 April 2019 Liver function indicators: ALT/AST 63/55 U/L, 10 May 2019 Normal thyroid function indicators: TSH, fT4, fT3, liver function indicators: ALT/AST/γ-GT 74/43/119U/L, 28 May 2019 Blood endocrine indicators: TSH: 0.080 Uiu/ml, fT4, fT3, ACTH: 2.8 pg/ml, Cortisol: 33.2 nmol/L, anti-SS-A antibody (+), 28 May 2019 tumor evaluation is based on the RECIST 1.1 (Response Evaluation Criteria in Solid Tumors 1.1) standard, and the evaluation effect is SD (stable disease). 31 May 2019 Enhanced MRI of the pituitary gland showed no abnormalities and mild sphenoid sinusitis (**Figure S1**). 4 June 2019 Liver function indicators are ALT/γ-GT 61/70 U/L, TT3 1.1 nM, TSH 0.120 uIU/ml, ACTH <1.5 pg/ml, cortisol: 19.5 nmol/L and FT3, TT4, FT4, Testosterone and growth hormone are in the normal range. 18 June 2019 Liver function indicators are ALT/γ-GT 67/133 U/L, TT3 1.0 nM, TSH 0.220 uIU/ml and FT3, TT4, FT4 are all within the normal range. 9 July 2019 ALT/AST 212/100 U/L, 16 July 2019 ACTH: 2.0 pg/ml, cortisol: 10.0 nmol/L, thyroid function indicators: in the normal range. Antibodies to hepatitis A, hepatitis C, and hepatitis E were all negative. 23 July 2019 ALT/AST/γ-GT 170/63/163 U/L, 31 July 2019 liver puncture guided by B-ultrasound, pathology: (liver puncture) morphology features suggested immune-related hepatitis (**Figure 2** and **Table 1**).

**Table 1:** The hormone levels of this patient in various stages.

	TT3	TT4	FT3	FT4	TSH	ACTH	COR	PTH
11 April 2019	/	/	3.9	15.7	1.19	/	/	/
10 May 2019	1.7	94.5	4.6	15	1.14	/	/	/
28 May 2019	/	/	4.5	12.9	0.08	2.8	33.2	47
4 June 2019	1.1	117	3.6	21.5	0.12	<1.5	19.5	/
18 June 2019	1	72.2	3.1	14.2	0.22	/	/	/
9 July 2019	/	/	4.5	16.3	2.99	/	/	/
30 July 2019	0.7	73	2.4	16.1	0.19	<1.5	36.9	/
10 September 2019	1.3	117	3.7	18.8	1.02	/	/	/
Normal range	1.3-3.1nmol/L	66-181nmol/L	2.8-7.1pmol/L	12-22pmol/L	0.27-4.2uIU/L	7.2-63.3 pg/mL	133.0-537.0 nmol/L	15-65pg/mL
	**LH**	**FSH**	**PRL**	**E2**	**PRG**	**TTE**		**GH**	
11 April 2019	/	/	/	/	/	/		/	
10 May 2019	/	/	/	/	/	/		/	
28 May 2019	/	/	/	/	/	/		/	
4 June 2019	14.6	19.9	294	<18.4	<0.159	7.6		0.1	
18 June 2019	/	/	/	/	/	/		/	
9 July 2019	/	/	/	/	/	/		/	
30 July 2019	/	/	/	/	/	/		/	
10 September 2019	/	/	/	/	/	/		/	
Normal range	1.7-8.6mIU/mL	1.5-12.4mIU/mL	131-647mIU/L	94.8-223pmol/L	<0.159nmol/L	9.9-27.8nmol/L		0-1ng/ml	

## Diagnosis

Combined with the clinical manifestations, physical examination and laboratory examinations of the patient, he was clinically diagnosed as grade 2 hypophysitis on 28 May 2019 with head, face and neck swelling, lower cortisol and ACTH value than normal, which is considered as an irAE. Meanwhile, his clinical treatment was suspended. On 31 July 2019, it was proved to be a manifestation of immune-related hepatitis pathologically by liver puncture. Considering the too long toxic and side effects, the patient got out of this clinical trial.

## Follow-Up Treatment

He started oral prednisolone acetate 20 mg tid on 28 May 2019. Facial swelling was significantly alleviated after 3 days of oral administration, and then the dose of glucocorticoid was gradually reduced. From 6 July 2019, the dose of prednisolone acetate was reduced to 10 mg qd PO. On 9 July 2019, ALT/AST is 212/100 U/L. On 16 July 2019, ACTH is 2.0 pg/ml and cortisol is 10.0 nmol/L. Thyroid function indicators are in normal range. Liver puncture on 31 July 2019 confirmed the manifestation of immune-related hepatitis pathologically (**Figure 2**).

## The Treatment of Complications Caused by Long-Term Oral Administration of Glucocorticoids

**Figure 3:**
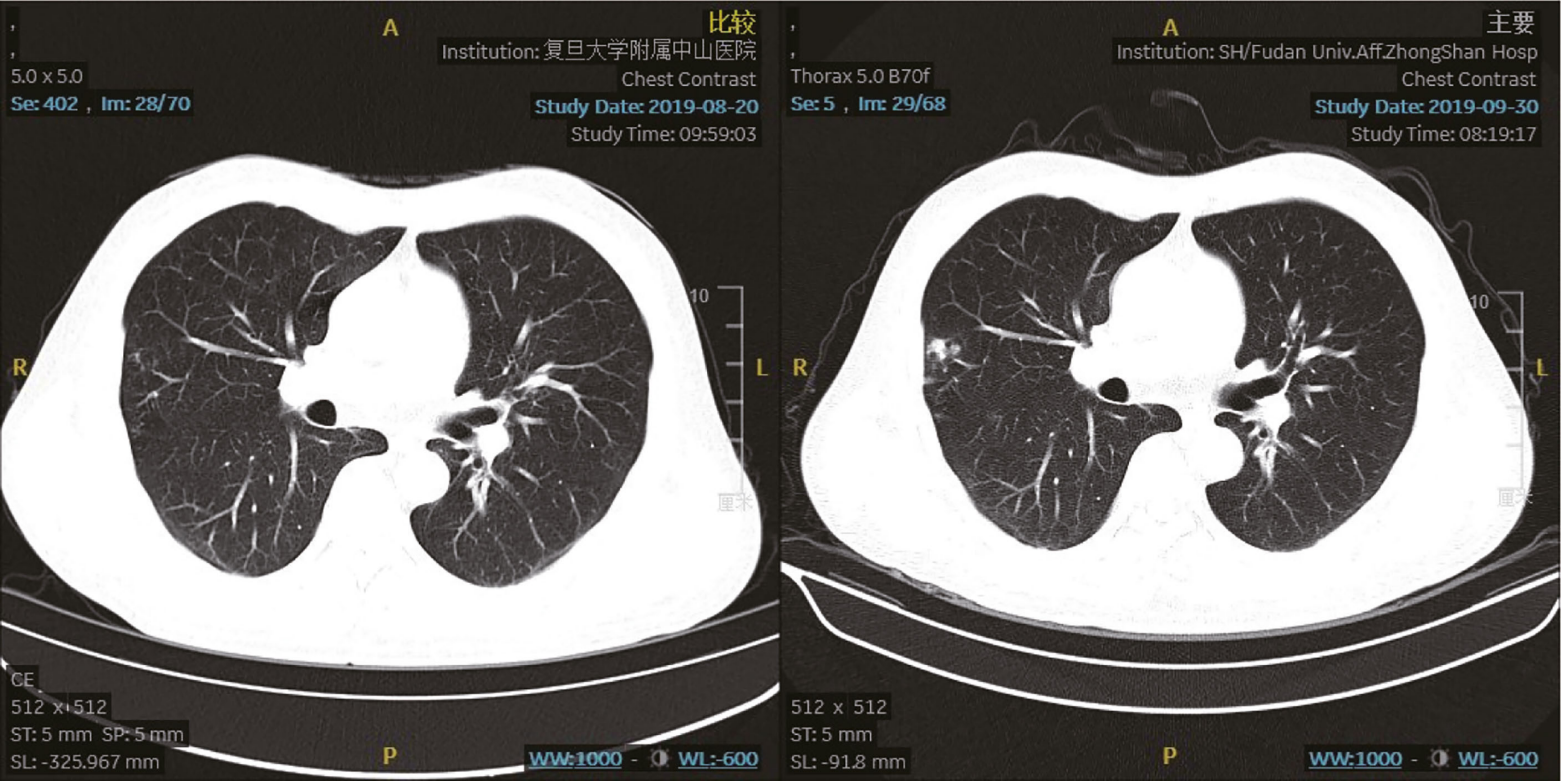
Chest computed tomography scan. The chest computed tomography lung window (left picture 20 August 2019; right picture 30 September 2019) shows new nodules on the lateral edge of the right lung, which may suggest inflammatory.

The prednisolone acetate dose of the patient was reduced to 10 mg qd P-O on 30 August 2019. On 13 August 2019, he felt abdominal distension and the skin on the right lower abdomen showed a band-like tingling sensation accompanied by itching and also decreased appetite, constipation and fatigue. Physical examination: ECOG (Eastern Cooperative Oncology Group) score 1, abdominal swelling, percussion drum sounds, pinpoint papules in the right lower abdomen, slightly higher local skin temperature and the lower limbs without swollen. On 16 August 2019, the dermatologist diagnosed this illness as “herpes zoster”. The patient was treated with antiviral, analgesic, and vitamin supplementation. At the same time, glucocorticoids were suspended. Liver function is under close follow-up. The herpes subsided a week later, but the pain persisted and was treated with analgesics. With follow-up CT on 30 September 2019, the tumor assessment is still SD. Chest CT showed that there were new nodules on the lateral edge of the right lung, possibly inflammatory (**Figure 3**). The blood T-SPOT was positive, and CT-guided puncture of the right lung nodule was performed on 8 October 8 2019. Postoperative pathology showed granulomatous lesions with coagulative necrosis. Acid-fast staining showed 2 positive bacteria, so mycobacteria could not be excluded. But the PCR test result was negative. Considering that the patient still has grade 2 immune liver damage (ALT/γ-GT 156/323 U/L, AST in normal range), amikacin 0.6 g qd vgtt + levofloxacin 0.4 g qd PO + ethamidine alcohol 0.75 g qd PO will be given from 11 October 2019.

**Figure 4:**
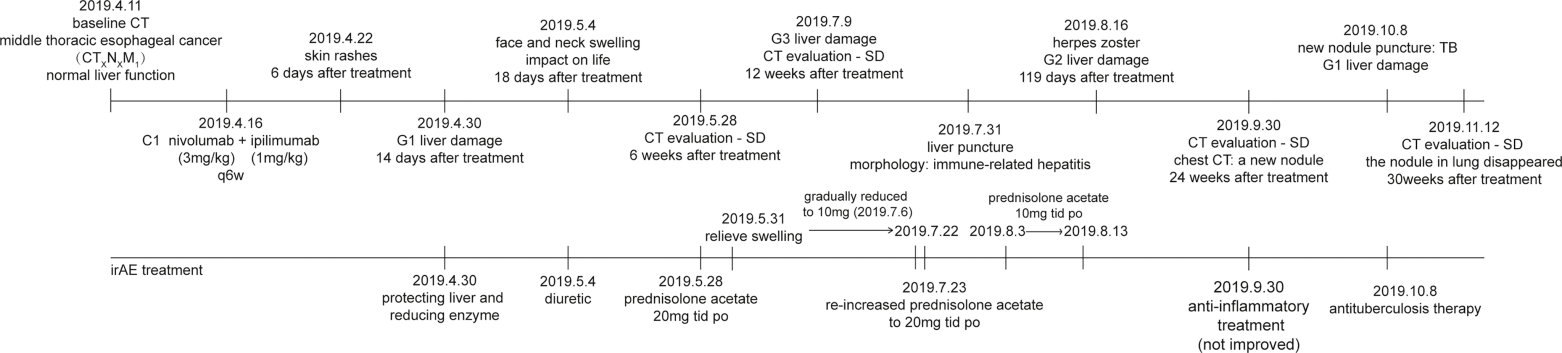
Timeline of interventions and outcomes.

We plotted the patient timeline to summary the entire treatment (**Figure 4**).

## Discussion

The binding of CTLA-4, PD-1 ligand and its receptor plays an important role in the suppression of the immune system. Ipilimumab is a monoclonal antibody that targets the CTLA-4 receptor and prevents its interaction with CD80/CD86 (4). Inhibition of CTLA-4 receptors has been proven to enhance the activation and proliferation of T cells, while also reducing the function of T-reg cells so that the immune system can better dominate the tumor (4). Otherwise, nivolumab is a monoclonal antibody directed at blocking the PD-1 receptor. The mechanism is to combine competitively with PD-1 ligands such as PD-L1 and PD-L2 to liberate the immune response suppressed by the PD-1 pathway (5). Nivolumab has been proven to enhance the anti-tumor effect of the immune system and reduce the tumor growth (5). Nivolumab combined with ipilimumab could cause stronger T cell activation and anti-tumor effects in certain cancer types than single therapy. However, the risk of adverse reactions of combination therapy is also greater than that of single-agent therapy (6).

Immune-related hepatitis and hypophysitis are two common adverse reactions caused by immune checkpoint inhibitors. The case discussed in this article was diagnosed by doing specific work-up to investigate the cause of liver injury including autoantibodies, serum gammaglobulin levels as same as these reporters described (7, 8). However, its occurrence is particularity due to the simultaneous onset of two immune-related adverse reactions and the recurrence of viral infection after hormone therapy. Similar cases have not been reported according to the results of the literature search. The 2019 National Cancer Comprehensive Network (NCCN) guidelines on immunotherapy-related toxicity management still proposed that glucocorticoids are the preference for the treatment of immune-related adverse reactions. In the treatment of pituitary inflammation caused by immune checkpoint inhibitors, if the patient has related symptoms, hormonal replacement therapy should be used. However, when neurological symptoms such as visual disturbances are present, high doses of glucocorticoids should be used and immunotherapy should be suspended until the acute symptoms of the patient are eliminated (1, 9). Also, another report described that the early treatment of high-dose glucocorticoid for irAE is associated with poorer prognosis in advanced melanoma (10). So, it is important to measure the state of disease and give the correct treatment strategy.

Correspondingly, in the treatment of hepatitis caused by immune checkpoint inhibitors, the NCCN guidelines also pointed out that glucocorticoids are the most common treatment option, but the specific dosage is not mentioned in the guidelines. A recent study showed that in the treatment of hepatitis caused by immune checkpoint inhibitors, customized therapy based on the own condition of the patient may be more effective than systemic treatment with glucocorticoids (10). When diagnosing hepatitis caused by immune checkpoint inhibitors, it is usually difficult to distinguish it from immune hepatitis based on the symptoms and signs of the patient, but histological and imaging results could help to do it (11, 12). A recent study summarized the histological characteristics of hepatitis caused by CTLA-4 inhibitors and PD-1 inhibitors. This information is helpful for doctors to find out the specific drugs that cause adverse reactions when immune checkpoint inhibitors combination, so that the treatment becomes more specific (13).

The results of the CheckMate 648 clinical study have been published in the New England Journal of Medicine since February 2022. Although the study was not designed to compare the efficacy between nivolumab plus ipilimumab and nivolumab plus chemotherapy, we could also find that the treatment with nivolumab plus ipilimumab provided better survival benefits for patients and the rate of adverse events did not increase significantly. In addition, treatment with nivolumab plus ipilimumab resulted in a longer median duration of response (11.8 months) than treatment with nivolumab plus chemotherapy (8.2 months) or chemotherapy alone (5.7 months). Nivolumab plus ipilimumab therapy may offer a new option for advanced esophageal squamous-cell carcinoma patients (14). Therefore, it is particularly important to carry out reasonable management and treatment for the possible side effects of immunotherapy combination.

In the case discussed in this article, the resurgence of tuberculosis and the onset of herpes zoster occurred three months after the patient received prednisolone treatment. Based on the past medical history of tuberculosis of the patient, the recurrence of tuberculosis after receiving long-term glucocorticoid therapy is expected. However, the patient has not previously reported any history of varicella infection or close contact with varicella-infected patients, so we did not expect the resurgence of herpes zoster virus during the treatment. This case emphasizes the importance of comprehensive collection of the past medical history upon admission of the patient. In addition, multiple case reports mentioned that patients treated with PD-1 inhibitors are at risk of developing tuberculosis infection (15–17). Therefore, physicians should be vigilant about possible opportunistic infections when using immunotherapy, regardless of whether the patient has a history of previous infections.

Furthermore, it is important to study the mechanism of irAE. T cells, B cells and antigen presenting cells (APCs) are all enrolled in the occurrence of irAE (18). Otherwise, Tregs are also the important subgroup for the balance of immune function. The inhibition of Tregs may contribute to the irAE (18, 19). The discussion of the mechanism could help researchers find a new way to avoid the occurrence of irAE.

## Conclusion

Combination therapy with CTLA-4 and PD-1 immune checkpoint inhibitors has a higher risk of adverse reactions than monotherapy. Patients who develop multiple adverse reactions to immunotherapy at the same time usually receive higher doses and longer courses of glucocorticoid therapy, which greatly increases the risk of complications such as the resurgence of the virus in this case. The current guidelines lack information on the adverse reactions of multiple immune treatment and complications caused by the treatment of their adverse reactions. This case emphasizes the importance of early diagnosis and intervention in patients with multiple immune-related adverse reactions. It opens a window for subsequent studies on immune-related adverse reactions and complications. Follow-up research is essential for the systematic management of multiple immune-related adverse reactions.

## Data Availability Statement

The raw data supporting the conclusions of this article will be made available by the authors, without undue reservation.

## Ethics Statement

The studies involving human participants were reviewed and approved by the Ethics Committee of Zhongshan Hospital, Fudan University. The patients/participants provided their written informed consent to participate in this study. Written informed consent was obtained from the individual(s) for the publication of any potentially identifiable images or data included in this article.

## Author Contributions

YF participates in all aspects of the center’s research as Sub-I and is familiar with the screening, treatment, efficacy, adverse reactions and outcomes of each selected patient. YF also wrote this manuscript. CL is a doctor of pharmacy. He gave professional opinions and analysis of possible causes in pharmacy on the multiple immune-related adverse reactions that occurred after the patient’s dual-immune treatment. YJ is a pathologist in our hospital, specializing in pathological genetic diagnosis and pathological manifestations of adverse immune reactions, providing a very professional analysis for the patient’s pathological diagnosis. YL is a CRC in our center, assisting my work and follows up the patient for a long time after drug withdrawal. Both doctors LG and YY gave me a lot of assistance during the diagnosis and treatment of the patient during the hospitalization after the occurrence of irAE. As the PI of the branch center, the corresponding author, TL gave me the most professional guidance and help during the entire research process and controlled the progress of the entire research project in our center. All authors listed have made a substantial, direct, and intellectual contribution to the work and approved it for publication.

## Conflict of Interest

Author YL is employed by Hangzhou Simo Co., Ltd.

The remaining authors declare that the research was conducted in the absence of any commercial or financial relationships that could be construed as a potential conflict of interest.

## Publisher’s Note

All claims expressed in this article are solely those of the authors and do not necessarily represent those of their affiliated organizations, or those of the publisher, the editors and the reviewers. Any product that may be evaluated in this article, or claim that may be made by its manufacturer, is not guaranteed or endorsed by the publisher.
