# Supplementary figures and images for: Nivolumab Combined With Ipilimumab Treatment Induced Hypophysitis and Immune-Mediated Liver Injury in Advanced Esophageal Squamous Cell Carcinoma: A Case Report
Source: Front Oncol. 2022 Apr 1;12:801924. doi: 10.3389/fonc.2022.801924 (PMC9012136; doi:10.3389/fonc.2022.801924)

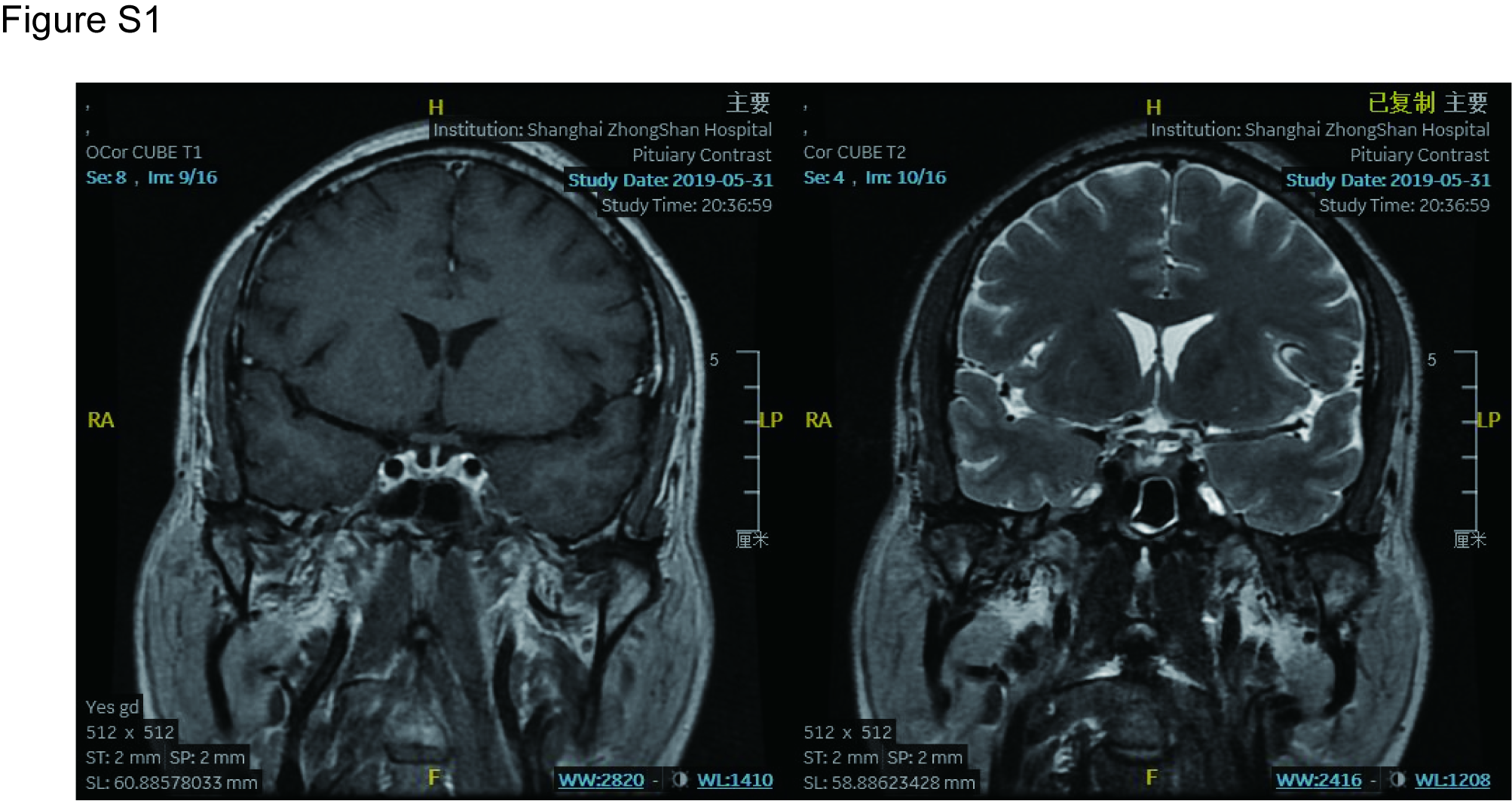

Supplement: Supplementary file 1 [file Image_1.tif]
